# Dickkopf-1 is regulated by the mevalonate pathway in breast cancer

**DOI:** 10.1186/bcr3616

**Published:** 2014-02-14

**Authors:** Tilman D Rachner, Andy Göbel, Stefanie Thiele, Martina Rauner, Peggy Benad-Mehner, Peyman Hadji, Thomas Bauer, Michael H Muders, Gustavo B Baretton, Franz Jakob, Regina Ebert, Martin Bornhäuser, Christian Schem, Lorenz C Hofbauer

**Affiliations:** 1 Division of Endocrinology and Metabolic Bone Diseases, Department of Medicine III, Technical University, Fetscherstr. 74, 01307 Dresden, Germany; 2 Department of Gynecology and Oncology, Philipps-University, Baldingerstr, 35034 Marburg, Germany; 3 Institute of Pathology, Technical University, Fetscherstr. 74, 01307 Dresden, Germany; 4 Orthopedic Center for Musculoskeletal Research, University of Würzburg, Brettreichstraße 11, 97074 Würzburg, Germany; 5 Division of Hematology, Department of Medicine I, Technical University, Fetscherstr. 74, 01307 Dresden, Germany; 6 Center for Regenerative Therapies Dresden, Technical University, Fetscherstr. 105, 01307 Dresden, Germany; 7 Department of Gynecology and Obstetrics, University Medical Center Schleswig-Holstein, Arnold-Heller-Straße 3, 24105 Kiel, Germany

## Abstract

**Introduction:**

Amino-bisphosphonates and statins inhibit the mevalonate pathway, and may exert anti-tumor effects. The Wnt inhibitor dickkopf-1 (DKK-1) promotes osteolytic bone lesions by inhibiting osteoblast functions and has been implicated as an adverse marker in multiple cancers. We assessed the effects of mevalonate pathway inhibition on DKK-1 expression in osteotropic breast cancer.

**Methods:**

Regulation of DKK-1 by bisphosphonates and statins was assessed in human breast cancer cell lines, and the role of the mevalonate pathway and downstream targets was analyzed. Moreover, the potential of breast cancer cells to modulate osteoblastogenesis via DKK-1 was studied in mC2C12 cells. Clinical relevance was validated by analyzing DKK-1 expression in the tissue and serum of women with breast cancer exposed to bisphosphonates.

**Results:**

DKK-1 was highly expressed in receptor-negative breast cancer cell lines. Patients with receptor-negative tumors displayed elevated levels of DKK-1 at the tissue and serum level compared to healthy controls. Zoledronic acid and atorvastatin potently suppressed DKK-1 *in vitro* by inhibiting geranylgeranylation of CDC42 and Rho. Regulation of DKK-1 was strongest in osteolytic breast cancer cell lines with abundant DKK-1 expression. Suppression of DKK-1 inhibited the ability of breast cancer cells to block WNT3A-induced production of alkaline phosphates and bone-protective osteoprotegerin in preosteoblastic C2C12 cells. In line with the *in vitro* data, treatment of breast cancer patients with zoledronic acid decreased DKK-1 levels by a mean of 60% after 12 months of treatment.

**Conclusion:**

DKK-1 is a novel target of the mevalonate pathway that is suppressed by zoledronic acid and atorvastatin in breast cancer.

## Introduction

Bone metastases remain a serious long-term complication in patients suffering from breast cancer and prostate cancer. Bisphosphonates (BPs) are established antiresorptive agents for the treatment of bone metastases. More recently, direct antitumor effects of BPs have been suggested, including induction of apoptosis and inhibition of migration, invasion, and (neo) vascularization [1]. BPs with antitumor activity are generally nitrogen-containing BPs that inhibit farnesyl pyrophosphate synthase, a key enzyme of the mevalonate pathway [2,3]. Statins are a second group of clinically established agents that inhibit the mevalonate pathway and have also been associated with an antitumor potential [4]. Hence, a number of clinical trials were initiated to investigate whether these promising preclinical results would translate into clinical efficacy for women with nonmetastatic breast cancer. Although modestly favoring the adjuvant use of bisphosphonates, these trials failed to provide a clear evidence for their general efficacy in adjuvant therapy. Of note, positive results were repeatedly reported for women with deprived estrogen levels as a result of menopause or hormone ablation therapy [5-8].

Dickkopf-1 (DKK-1), a soluble inhibitor of the canonical Wnt signaling pathway, has been linked to osteolytic bone disease [9]. In multiple myeloma, increased levels of DKK-1 are associated with the presence of lytic bone lesions [10]. DKK-1 is thought to promote osteolytic bone disease directly by inhibition of osteoblasts and concurrently promoting osteoclasts via suppression of osteoprotegerin (OPG) and enhancing receptor activator of nuclear factor-κB ligand [11]. In murine models of myeloma, inhibition of DKK-1 reduced the extent of osteolytic lesions [12,13]. In contrast to myeloma bone disease, the role of DKK-1 in breast cancer and prostate cancer is less clear. In metastatic breast cancer, serum levels of DKK-1 are increased [14], and breast cancer-derived DKK-1 has the ability to inhibit osteoblast differentiation [15]. In women with triple-negative breast cancer, which is associated with a high risk of recurrence, expression of DKK-1 indicated poor outcome of patients [16]. In prostate cancer, DKK-1 expression increases in early stages while decreasing during progression towards metastatic disease [17]. These data indicate that DKK-1 may have a pathophysiological role in skeletal metastases of breast and prostate cancer. Here we identified DKK-1 as a novel target of the mevalonate pathway in estrogen receptor (ER)-negative breast cancer that can be modulated by BPs and statins via inhibiting of geranylgeranylation.

## Methods

### Cells

Breast cancer cells (MDA-MB-231, MCF-7 and T47D) and prostate cancer cells (PC-3 and MDA-PCa2b) were purchased from ATCC (Manassas, VA, USA), except for MDA-BONE cells (also known as MB-231-TxSA) that were obtained from the University of Texas (San Antonio, TX, USA) and MDA-MET cells that were a kind gift from Prof. L Suva (Center for Orthopaedic Research, University of Arkansas AR, USA). For osteoblast experiments, the murine myoblast cell line mC2C12 was used. C2C12 cells are a murine myoblast cell line capable of differentiation towards osteoblasts in the presence of Wnt ligands. Murine C2C12, control L-cells and WNT3A-L-Cells were a kind gift from Dr Michael Stock (University of Erlangen, Germany). Prostate cancer cell lines were kept in RPMI 1640 medium from Bio West (Renningen, Germany). All other cell lines were cultured in Dulbecco’s modified Eagle’s medium/Ham’s F-12 (PAA, Pasching, Austria), 10% fetal calf serum supreme (Lonza, Cologne, Germany) and 1% penicillin/streptomycin (PAA). Human microvascular endothelial cells-1 were cultured in endothelial cell growth medium 2 (PromoCell, Heidelberg, Germany). Primary cultures of human umbilical vein endothelial cells were isolated using collagenase II as described previously [18]. All cell lines that were not purchased from ATCC were obtained following the ethical guidelines of our institution and the providing institution. Cell lines were sent to DSMZ (German Collection of Microorganisms and Cell Culturs), where authenticity was determined by short tandem repeat profiling and by matching with the known profiles.

### Small interfering RNA, antibodies and reagents

Zoledronic acid was provided by Novartis (Basel, Switzerland), and atorvastatin, mevalonate, geranyl-geranyl-pyrophosphate (GGPP), farnesyl pyrophosphate (FPP), GGTI-298 and FTI-277 were obtained from Sigma-Aldrich (Munich, Germany). Rac1 inhibitors #1 (553502) and #2 (553511), Y-27632, rho kinase inhibitor (H-1152P) and *Clostridium difficile* toxin A were from Merck Chemicals (Darmstadt, Germany). Cdc42 inhibitor ML-141 was from Tocris Bioscience (Bristol, UK). Rho inhibitor #2 (BML-EI394) was obtained from Enzo (Lörrach, Germany). Rho inhibitor #1 (CT04) and Rho/Rac/Cdc42 activator I were from Cytoskeleton Inc. (Denver, CO, USA). Recombinant human DKK-1 was obtained from Peprotech (Hamburg, Germany). Antibody for RAP1A (sc-1482) was from Santa Cruz (Heidelberg, Germany), RAS (610001) antibody was from BD Biosciences (Heidelberg, Germany), the DKK-1 (MAB10962) antibody was from R&D Systems (Wiesbaden, Germany) and all other antibodies were obtained from Cell Signaling Technology (Frankfurt, Germany). DKK-1 small interfering RNA (siRNA; s22721 and s22723), Cdc42 siRNAs and nontarget siRNA were purchased from Applied Biosystems (Darmstadt, Germany). Cells were transfected using Dharmafect (Thermo Scientific, Waltham, Massachusetts (MA) USA).

### RNA isolation, reverse transcription and real-time polymerase chain reaction

RNA from cell cultures was isolated using the HighPure RNA extraction kit from Roche Applied Science (Mannheim, Germany) according to the manufacturer’s protocol. RNA (500 ng) was reverse transcribed using Superscript II (Life Technologies, Darmstadt, Germany) and used for SYBR green-based real-time polymerase chain reaction (PCR) using a standard protocol (Applied Biosystems). Primer sequences were: hu glyceraldehyde 3-phosphate dehydrogenase (GAPDH) sense, AGCCACATCGCTCAGACAC; hu GAPDH antisense, GCCCAATACGACCAAATCC; hu Dkk-1 sense, AGCACCTTGGATGGGTATTC; hu DKK-1 antisense, CACACTTGACCTTCTTTCAGGAC; mu ACTB sense, GATCTGGCACCACACCTTCT; mu ACTB antisense, GGGGTGTTGAAGGTCTCAAA; mu ALP sense, CTGGTGGCATCTCGTTATCC; mu ALP antisense, CTACTTGTGTGGCGTGAAGG; and mu OPG sense, CCTTGCCCTGACCACTCTTA; mu OPG antisense, CCTTGCCCTGACCACTCTTA. PCR conditions were 50°C for 2 minutes and 95°C for 10 minutes followed by 40 cycles with 95°C for 15 seconds and 60°C for 1 minute. The melting curve as assessed in the following program: 95°C for 15 seconds, 60°C for 1 minute and 95°C for 30 seconds. The results were calculated applying the ΔΔCT method and are presented as the *x*-fold increase relative to the housekeeping gene (GAPDH or β-actin) or as a percentage of control.

### WNT array

The human WNT signaling pathway PCR array (PAHS-0437; QIAGEN, Hilden, Germany) was used to screen for Wnt-related targets and was performed using the provided protocols. Briefly, cDNA templates of zoledronic acid-treated and control-treated MDA-231 cells were mixed with the appropriate PCR master mix. Equal volumes were pipetted to each well of the same PCR array. PCRs were performed as described above. Assessment of three individual experiments was conducted using the software provided by the manufacturer.

### cDNA polymerase chain reaction array

The breast cancer cDNA Array II was purchased from Origene (Rockville, MD, USA) and was assessed for DKK-1 expression, normalized to GAPDH using the supplier’s protocol. This array contains 48 samples, covering five normal breast tissues and 43 samples of breast cancer, and provides clinical information including hormone status and pathological grade.

### Dickkopf-1 and osteoprotegerin enzyme-linked immunosorbent assay and serum markers of bone turnover

Human DKK-1 and murine OPG enzyme-linked immunosorbent assays were obtained from Biomedica (Vienna, Austria) and were performed according to the manufacturer’s instructions. Cell supernatants were prediluted as determined by pretesting. Serum samples of breast cancer patients and healthy controls were obtained after informed consent and Institutional Review Board approval. Details of patient characteristics are shown in Figure S3B in Additional file 1. Patients were women with hormone-negative, nonmetastatic, breast cancer who were blinded to receive infusions of either 4 mg zoledronic acid or placebo every 3 months as adjuvant therapy. These patients received 1,000 mg calcium and 1,000 IU vitamin D per day for the duration of the study after Institutional Review Board approval (97/05 (A)/KKS 1009 and informed patient consent had been obtained. Detailed patient characteristics are shown in Figure S4A in Additional file 1. No additional concomitant drugs known to influence bone turnover were given. Control serum was taken before the first administration of zoledronic acid and before each further administration. Blood samples were immediately worked up and stored at -80°C until the analyses were performed. Markers of bone turnover were measured as routine parameters by our clinical laboratory.

### Immunoblotting

Western blot analyses were performed as described previously [19]. Briefly, after completion of the experiments, cells were lysed and quantified. In general, 20 μg protein were loaded for SDS-PAGE and transferred onto a 0.2 μm nitrocellulose membrane. After blocking for 1 hour with 5% nonfat dry milk in Tris-buffered saline with 1% Tween-20, membranes were incubated with a primary antibody overnight. After washing, the membrane was incubated for 1 hour with the horseradish peroxidase-conjugated secondary antibody. Membranes were then washed three times with Tris-buffered saline with 1% Tween-20, and proteins were visualized with Super Signal (Pierce, Bonn, Germany) enhanced chemiluminescence.

### Immunohistochemistry and quantitative assessment

Primary breast cancer tissue was assessed using immunohistochemistry. Multiple tissue microarrays (TMA 8501) were purchased from Tristar (Rockville, MD, USA). Paraffin-embedded sections were dewaxed, rehydrated using an alcohol gradient, and heat-retrieved from antigens. Endogenous peroxidase activity was blocked using 0.3% H_2_O_2_/phosphate-buffered saline for 10 minutes at room temperature and nonspecific binding sites using the blocking buffer of the VECTASTAIN Elite ABC Kit (VECTOR Laboratories, Peterborough, UK) for 45 minutes at room temperature. Afterwards, sections were incubated with a polyclonal anti-DKK-1 antibody (ab22827; Abcam, Milton, UK) overnight at 4°C. Subsequently, slides were treated with an anti-goat secondary antibody conjugated to biotin and then developed utilizing avidin-conjugated horseradish peroxidase with diaminobenzidine as substrate (DAKO, Hamburg, Germany). Specificity was validated in cell sections where DKK-1 levels were diminished using siRNA (Figure S3A in Additional file 1). Staining intensity was assessed by two individual and experienced pathologists (MHM and GBB) and rated as either none (0), weak (1), moderate (2) or strong (3). Interobserver variability was measured using the Cohen’s κ test.

### Statistical analysis

Results are presented as the mean ± standard deviation. All experiments were repeated at least three times. Statistical evaluations were performed using a one-way analysis of variance or Student’s *t* test. *P* < 0.05 was considered statistically significant.

## Results

### Dickkopf-1 expression is suppressed by zoledronic acid and atorvastatin in breast cancer cell lines *in vitro*

**Figure 1:**
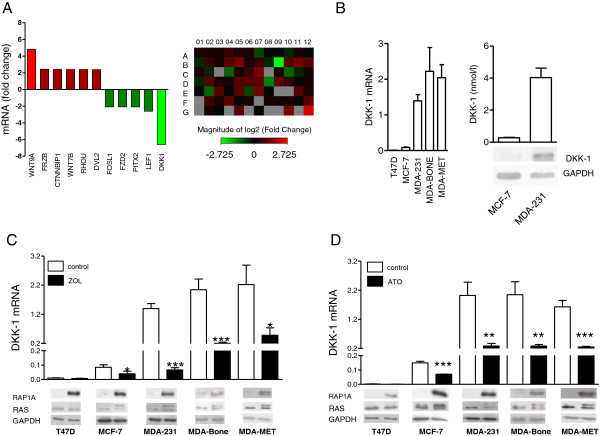
**Dickkopf-1 is a target of zoledronic acid and atorvastatin in breast cancer cells *in vitro*. (A)** Results from a WNT PCR array define Dickkopf-1 (DKK-1) as a target of zoledronic acid. MDA-231 cells were treated with zoledronic acid (100 μM) for 24 hours and assessed by a WNT array. A heat map of all tested genes is shown on the right: green, increased expression compared with control; red, decreased expression compared with control; grey, nondetectable gene. All genes that were regulated at least twofold are separately depicted on the left. **(B)** Basal expression of DKK-1 in different breast cancer cell lines assessed by polymerase chain reaction (PCR), enzyme-linked immunosorbent assay (ELISA) and western Blot. Glyceraldehyde 3-phosphate dehydrogenase (GAPDH) is a loading control. **(C)**, **(D)** Suppression of DKK-1 by zoledronic acid (ZOL; 100 μM) and atorvastatin (ATO; 10 μM) after 24 hours of exposure was confirmed in multiple cell lines. Assessment of unfarnesylated RAS (upper band) and ungeranylated RAP1A confirmed mevalonate pathway inhibition. Representative blots are shown. GAPDH is used as a loading control. Data for PCR and ELISA are shown as mean ± standard deviation of three independent experiments. *P < 0.05; **P < 0.01; ***P < 0.001.

To identify novel targets of zoledronic acid, a Wnt signaling pathway PCR array was conducted with MDA-231 breast cancer cells after 24 hours of treatment with zoledronic acid. Eleven genes were regulated more than twofold (Figure 1A; full list of genes shown in Figure S1A in Additional file 1). DKK-1 was the utmost regulated gene, with a sevenfold suppression. To further assess the underlying mechanism(s) of this observation, baseline expression of DKK-1 was assessed in multiple breast cancer and prostate cancer cell lines. Overall, DKK-1 expression was detectable in all cell lines tested. Intriguingly, expression of DKK-1 was approximately 10-fold to 100-fold higher in the ER-negative and highly metastatic MDA-231, MDA-BONE and MDA-MET cell lines, compared with ER-positive MCF-7 and T47D cells (Figure 1B).

Next, suppression of DKK-1 by zoledronic acid was confirmed in these cell lines. The most potent inhibitory effects of DKK-1 by zoledronic acid (100 μM) were observed in those cell lines with the highest constitutive expression of DKK-1 (Figure 1C). Successful blockade of the mevalonate pathway was confirmed by western blot detection of unprenylated RAS and RAP1A, which are exclusively farnesylated (RAS) and geranylated (RAP1A) downstream targets of the mevalonate pathway.

The same cell lines were then exposed to 10 μM atorvastatin for 24 hours, which resulted in a comparable accumulation of unprenylated RAS and RAP1A, indicating potent inhibition of the mevalonate pathway. Atorvastatin reduced DKK-1 expression as seen previously with zoledronic acid (Figure 1D). Of note, zoledronic acid and atorvastatin failed to reduce DKK-1 expression in ER-positive T47D cells, the cell line that also displayed the lowest basal DKK-1 levels.

Both zoledronic acid and atorvastatin caused a dose-dependent inhibition of DKK-1 in MDA-231 cells (Figure S2A,B in Additional file 1). In zoledronic acid-treated cells, modest effects were seen starting at 10 μM. In the case of atorvastatin, concentrations of 1 μM were sufficient to significantly suppress DKK-1 (*P* < 0.01). Interestingly, 2-hour exposure to zoledronic acid resulted in a reduction of DKK-1 24 hours later that was comparable with continuous exposure (Figure S2C in Additional file 1). We observed no effect in cells treated with atorvastatin following the same protocol (2-hour exposure, measurement 24 hours later), indicating different mechanisms of cellular uptake (data not shown).

To analyze whether suppression of DKK-1 is limited to breast cancer cells, we also assessed DKK-1 levels in prostate cancer cells and endothelial cells following exposure to zoledronic acid and atorvastatin. In line with the proposed effect of DKK-1 exposure on osteoblast inhibition, PC3 cells, which form osteolytic lesions *in vivo*, showed high baseline levels of DKK-1, whereas MDA-PCa 2b cells, which have an osteoblastic phenotype, had low DKK-1 expression (Figure S1B in Additional file 1). Treatment with zoledronic acid and atorvastatin significantly reduced DKK-1 expression levels in PC3 cells. Similar effects were seen in human microvascular endothelial cells-1 and human umbilical vein endothelial cells (Figure S1C,D in Additional file 1). These findings indicate that regulation of DKK-1 by BPs and statins is a more general effect that is not specific to breast cancer.

### Dickkopf-1 is overexpressed in estrogen receptor-negative primary breast cancer

**Figure 2:**
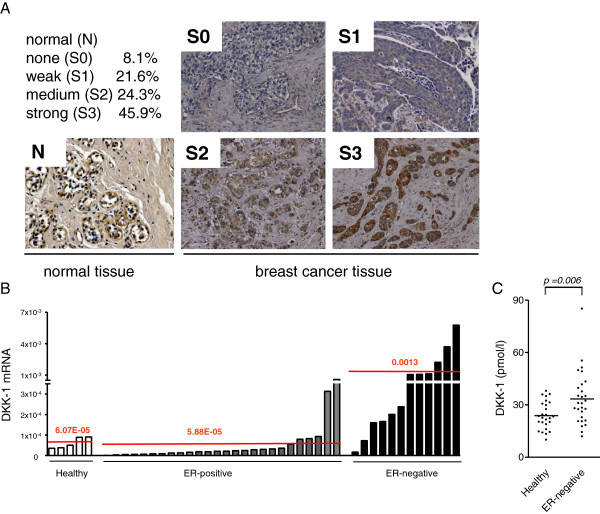
**Dickkopf-1 expression in breast cancer samples and serum. (A)** Dickkopf-1 (DKK-1) expression was assessed in a breast cancer tissue microarray consisting of 74 breast cancer samples and 10 normal tissues. The TMA was stained with an antibody directed against human DKK-1 and staining was graded from none (0) to strong (3). **(B)** A cDNA breast cancer array was used to quantify DKK-1 expression in breast cancer. Expression of DKK-1 was measured and normalized to β-actin. **(C)** DKK-1 serum levels are elevated (*P* = 0.006) in patients with estrogen receptor (ER)-negative breast cancer (*n* = 28) or in healthy controls (*n* = 27). Patients’ characteristics are listed in Figure S3B in Additional file 1.

Immunohistochemical staining of primary breast cancer tissue revealed a medium to strong expression of DKK-1 in 70% (52/74) of evaluated breast cancer cases, mainly in the cytoplasm. Of note, DKK-1 protein expression in normal breast tissue was limited to the myoepithelium surrounding the non-neoplastic glands (Figure 2A). Assessment of DKK-1 expression in a cohort of 43 breast cancer patients and five healthy controls showed increased expression of DKK-1 mRNA in many tumors compared with control, with no correlation between DKK-1 and tumor stage (data not shown). However, when correlated with the ER status, we found DKK-1 expression to be considerably higher in ER-negative tumors than in other samples (Figure 2B). While the mean expression of DKK-1 (relative to β-actin) was unchanged in patients with ER-positive tumors compared with normal breast tissue, it was increased by 21-fold in ER-negative samples. To test whether these findings translated into relevant changes at serum level, we measured DKK-1 levels in patients with ER-negative (nonmetastatic) breast cancer and compared them with a matched cohort (age, body mass index and menopausal status; Figure S3B in Additional file 1) of healthy controls. While healthy controls had mean DKK-1 serum levels of 23.8 pmol/l (*n* = 27), these levels significantly increased to 33.4 pmol/l (*n* = 28) in breast cancer patients (Figure 2C; *P* = 0.006; 95% confidence interval = -16.32 to -2.876).

### Dickkopf-1 is suppressed via inhibition of protein geranylgeranylation in breast cancer cell lines

**Figure 3:**
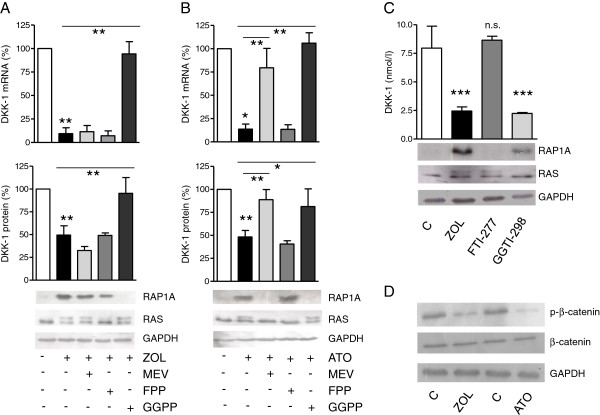
**Dickkopf-1 is regulated via inhibited geranylgeranylation. (A)**, **(B)** MDA-231 cells were treated with either zoledronic acid (ZOL; 100 μM) or atorvastatin (ATO; 10 μM) for 24 hours alone or concurrently with mevalonate substrates (geranyl-geranyl-pyrophosphate (GGPP), farnesyl pyrophosphate (FPP) or mevalonate (MEV)). Dickkopf-1 (DKK-1) was analyzed by polymerase chain reaction (PCR) and enzyme-linked immunosorbent assay (ELISA). **(C)** Specific inhibitors of geranylgeranylation GGTI-298 (5 μM) and farnesylation FTI-277 (100 nM) were used for further pathway clarification, and treatment was for 24 hours. Results of ELISA and PCR data are presented as the mean ± standard deviation of three independent experiments. *P < 0.05; **P < 0.01; ***P < 0.001. Inhibition of geranylgeranylation by GGTI-298, atorvastatin and zoledronic acid, farnesylation by FTI-277, atorvastatin and zoledronic acid as well as their selective reversal by the substrates was confirmed by assessing unfarnesylated RAS and ungeranylated RAP1A in western blot. Glyceraldehyde 3-phosphate dehydrogenase (GAPDH) is used as loading control. Representative blots are shown. **(D)** Levels of phosphorylated β-catenin decrease following 24 hours of exposure to zoledronic acid and atorvastatin. Levels of total β-catenin remain unchanged. GAPDH is used as a loading control.

We next confirmed whether the observed suppression of DKK-1 was directly mediated via inhibition of the mevalonate pathway. MDA-231 cells were treated with either zoledronic acid or atorvastatin and were co-treated with the mevalonate pathway substrates mevalonate, GGPP, or FPP. In the presence of mevalonate, the effects of atorvastatin but not of zoledronic acid on DKK-1 were completely reversed. Supplementation of GGPP fully restored expression of DKK-1 in atorvastatin-treated and zoledronic acid-treated cells, whereas FPP had no effect (Figure 3A,B). Substrates alone did not affect DKK-1 expression (data not shown). As GGPP restores geranylgeranylation of proteins and FPP restores farnesylation, which was confirmed by assessment of RAS and RAP1A (Figure 3A,B), these results imply that DKK-1 is regulated via inhibited geranylgeranylation. To further verify this hypothesis, cells were treated with FTI-277 or GGTI-298, selective inhibitors of farnesyltransferase or geranylgeranyltransferase I, respectively. Here, only treatment with GGTI (5 μM) significantly inhibited DKK-1 (Figure 3C). Effects were of comparable magnitude to zoledronic acid, and relevant inhibition of farnesyltransferase and geranylgeranyltransferase I was confirmed using western blot analyses of RAS and RAP1A. Of note, treatment with both zoledronic acid and atorvastatin decreased intracellular levels of phosphorylated β-catenin, an effect expected with decreasing DKK-1 levels and suggestive of increased Wnt signaling (Figure 3D).

### Cdc42 and Rho are involved in the regulation of Dickkopf-1

**Figure 4:**
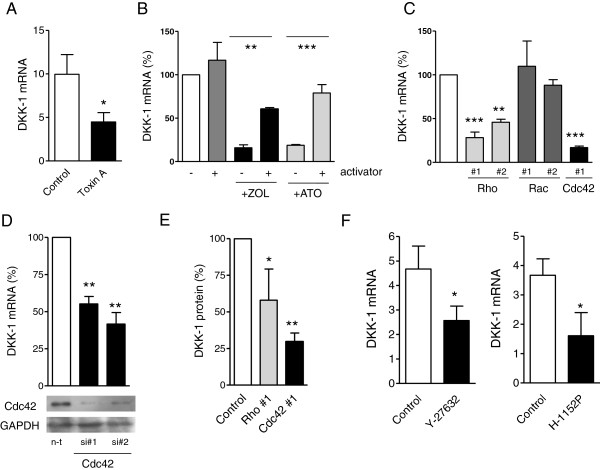
**Dickkopf-1 is regulated via Cdc42 and Rho in breast cancer. (A)** MDA-231 cells were treated for 48 hours with *Clostridium difficile* toxin A (250 ng/ml) and assessed for Dickkopf-1 (DKK-1) expression. **(B)** MDA-231 cells were exposed to zoledronic acid (ZOL) or atorvastatin (ATO) alone or concurrently with a Rho/Rac/Cdc42 activator for 24 hours. Due to the relatively short half-life of the activator, cells were treated every 5 hours with 1 μg/ml of activator until cells were harvested after 15 hours. **(C)** MDA-231 cells were treated with a number of different GTPase inhibitors for 24 hours and assessed for DKK-1 expression. Concentrations used were 10 μM for Rho#1 and Rho#2, 100 μM for Rac#1, 10 μM for Rac#2 and 50 μM for the Cdc42 inhibitor. **(D)** MDA-231 cells were transfected with control single interfering RNA (siRNA) or two different siRNAs directed against Cdc42. Sufficient knockdown was verified by western blot. DKK-1 expression was analyzed 48 hours after transfection. **(E)** Regulation of DKK-1 by Cdc42 and Rho was verified at protein levels, by measuring DKK-1 levels in the supernatant of MDA-231 cells treated with the same inhibitors as in C. **(F)** The role of Rho signaling was further confirmed by measuring DKK-1 levels following 24 hours of Rho-associated protein kinase inhibition using Y-27632 and H-1152P. Results of enzyme-linked immunosorbent assay and polymerase chain reaction data are presented as the mean ± standard deviation of three independent experiments. *P < 0.05; **P < 0.01; ***P < 0.001. GAPDH, glyceraldehyde 3-phosphate dehydrogenase.

*C. difficile* toxin A inhibits the GTPases Rho, Rac1 and Cdc42 [20]. To test whether these geranylgeranylated target proteins are involved in the regulation of DKK-1, MDA-231 cells were treated with toxin A. Treatment led to a significant inhibition of DKK-1 expression (Figure 4A). To further validate the role of GTPases in BP-induced and statin-induced DKK-1 suppression, cells were treated with either zoledronic acid or atorvastatin alone, or in combination with a GTPase activator (which activates Rho, Rac1 and Cdc42). As expected, single treatment with atorvastatin and zoledronic acid suppressed DKK-1 by >80% (*P* < 0.001). Activator treatment alone did not significantly affect DKK-1 expression, whereas simultaneous co-treatment with activator and atorvastatin or zoledronic acid largely restored DKK-1 levels in MDA-231 cells (*P* < 0.01). These results show that DKK-1 suppression is regulated via the GTPases Rho, Rac1 and/or Cdc42 (Figure 4B). Further differentiation between these GTPases was conducted using selective inhibitors. Here, inhibition of Cdc42 and Rho, but not Rac1, resulted in a significant suppression of DKK-1 expression and protein secretion (Figure 4C,D,E). Results were verified with two different inhibitors, or in the case of Cdc42 using an inhibitor and siRNA, and results were confirmed in MDA-bone cells (data not shown). In addition, treatment of cells with Rho-associated protein kinase inhibitors Y-27632 and H-1152P significantly decreased DKK-1 (Figure 4F). Taken together, these results suggest that mevalonate blockade suppresses DKK-1 via inhibited geranylgeranylation of Cdc42 and Rho.

### Zoledronic acid regulates alkaline phosphatase and osteoprotegerin in mC2C12 cells by inhibiting Dickkopf-1 *in vitro*

**Figure 5:**
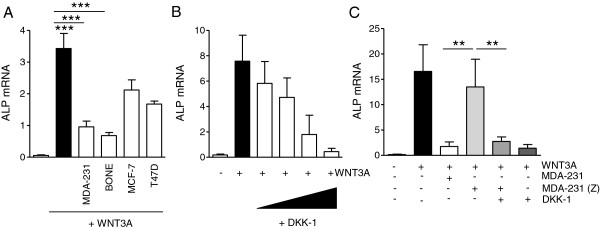
**Dickkopf-1 inhibits WNT3A-induced osteoblastic differentiation in C2C12 cells. (A)** Supernatants of breast cancer cells were harvested after 48 hours. C2C12 cells were cultured in control medium (containing 50% control L-cell medium) or differentiated in WNT3A-containing media (50%) and concurrently treated with breast cancer supernatants (25%) or unconditioned control media (25%). C2C12 cells were treated for 48 hours and assessed for alkaline phosphatase (ALP) expression. **(B)** C2C12 cells were differentiated with WNT3A media and exposed to increasing concentrations of recombinant Dickkopf-1 (DKK-1; 10, 50, 100 and 250 ng/ml). **(C)** C2C12 cells were exposed to supernatants of untreated or zoledronic acid-treated MDA-231 cells in the presence of WNT3A. To prevent potential direct effects of zoledronic acid on C2C12 cells, MDA-231 cells were preincubated with zoledronic acid for 24 hours, media were then replaced and zoledronic acid-free supernatants were harvested 48 hours later. Results of polymerase chain reaction data are presented as the mean ± standard deviation of three independent experiments. *P < 0.01; ***P < 0.001.

**Figure 6:**
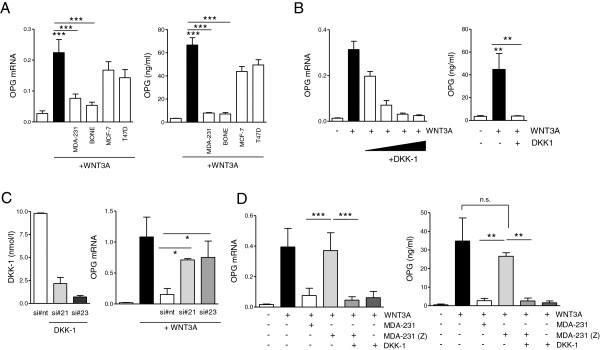
**Dickkopf-1 inhibits WNT3A-induced osteoprotegerin production in C2C12 cells. (A)** Supernatants of breast cancer cells were harvested after 48 hours. C2C12 cells were cultured in control medium (containing 50% control L-cell medium) or differentiated in WNT3A-containing media (50%) and concurrently treated with breast cancer supernatants (25%) or unconditioned control media (25%). C2C12 cells were treated for 48 hours and assessed for osteoprotegerin (OPG) expression and protein secretion. **(B)** C2C12 cells were differentiated with WNT3A media and exposed to increasing concentrations of recombinant Dickkopf-1 (DKK-1; 10, 50, 100 and 250 ng/ml). **(C)** DKK-1 was transiently knocked down in MDA-231 cells using two different single interfering RNAs (siRNAs). Knockdown was verified by quantifying DKK-1 levels secreted into the supernatant 48 hours after transfection. Supernatants of control siRNA and DKK-1 siRNA-treated cells were given to C2C12 cells during WNT3A-induced differentiation in the established ratios of 25% and 50%. OPG levels were determined after 48 hours of exposure. **(D)** C2C12 cells were exposed to supernatants of untreated or zoledronic acid-treated MDA-231 cells in the presence of WNT3A. To prevent potential direct effects of zoledronic acid on C2C12 cells, MDA-231 cells were preincubated with zoledronic acid for 24 hours, media were then replaced and zoledronic acid-free supernatants were harvested 48 hours later. Suppressed DKK-1 levels in the supernatant were confirmed by enzyme-linked immunosorbent assay (ELISA) prior to use. Results of ELISA and polymerase chain reaction data are presented as the mean ± standard deviation of three independent experiments. *P < 0.05; **P < 0.01; ***P < 0.001.

In the setting of bone metastases, DKK-1 is best known for its inhibitory effects on osteoblasts and thereby indirectly enhancing osteolytic bone destruction. In addition to its direct effects on osteoblasts, DKK-1 has also been reported to inhibit osteoblast-derived OPG production while promoting receptor activator of nuclear factor-κB ligand. We used C2C12 cells to investigate effects of DKK-1 modulation by zoledronic acid on alkaline phosphates (ALPs) and bone-protective OPG production. The presence of WNT3A-containing medium from WTN3A-overexpressing L-cells induced ALP and OPG production in C2C12 cells. When co-incubated with supernatants from different breast cancer cell lines, those with high baseline levels of DKK-1 inhibited ALP induction by WNT3A, whereas MCF-7 and T47D cells with low DKK-1 levels had a smaller effect (Figure 5A). Similarly, MCF-7 and T47D were unable to affect WNT3A-induced OPG production, whereas MDA-231 and MDA-BONE almost completely suppressed OPG (*P* < 0.001; Figure 6A). To demonstrate that WNT3A-mediated induction of an osteoblastic phenotype is effectively inhibited by DKK-1, C2C12 cells were treated with increasing concentrations of DKK-1 in the presence of WNT3A (Figures 5B and 6B). DKK-1 dose-dependently decreased ALP and OPG production. Lower doses of DKK-1 were required to inhibit OPG than ALP. Furthermore, knocking down DKK-1, as verified by PCR and enzyme-linked immunosorbent assay, diminished the ability of MDA-231 cells to inhibit WNT3A-induced OPG production (Figure 6C). Finally, MDA-231 cells pretreated with zoledronic acid failed to inhibit WNT3A-induced ALP and OPG production, an effect that was fully restored by supplementation of recombinant DKK-1 (Figures 5C and 6D).

### Dickkopf-1 serum levels decrease in breast cancer patients receiving adjuvant zoledronic acid

**Figure 7:**
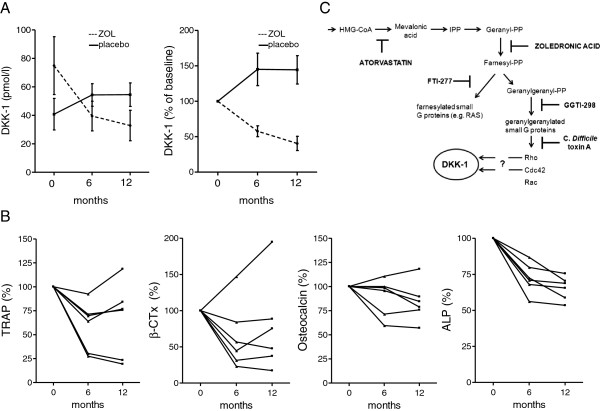
**Dickkopf-1 levels are suppressed in women with breast cancer following zoledronic acid. (A)** Dickkopf-1 (DKK-1) levels were measured in women with estrogen receptor (ER)-negative nonmetastatic breast cancer, who received 3-monthly infusions of zoledronic acid (ZOL; *n* = 6) or placebo (*n* = 3). DKK-1 levels are presented as mean absolute values and mean percentage of baseline expression. **(B)** Markers of bone turnover were assessed at the same time points as DKK-1. Values are presented as percentage of baseline expression. **(C)** Schematic regulation of DKK-1 by mevalonate pathway modulators via inhibition of Cdc42 and Rho. ALP, alkaline phosphatase; CTx, C-terminal telopeptide; IPP, isopentenyl pyrophosphate; TRAP, tartrate-resistant acid phosphatase; PP, pyrophosphate.

As a proof of principle that DKK-1 is a clinically relevant target of the mevalonate pathway, we assessed serum DKK-1 levels in patients with ER-negative and nonmetastatic breast cancer who received either 4 mg zoledronic acid (*n* = 6) or placebo (*n* = 3) every 3 months. All patients receiving zoledronic acid showed decreased DKK-1 levels at 6 and 12 months compared with baseline values. On average DKK-1 levels were decreased by 60% (± 22.7% standard deviation) at 12 months, compared with an increase of 44% (± 28% standard deviation) in the placebo group (Figure 7A; Figure S4B in Additional file 1). Absolute values of DKK-1 were widely spread, ranging from 13 to 133 pmol/l. Interestingly, patients with the highest baseline levels also showed the most rapid decline of DKK-1 levels after 6 months of treatment. We further assessed markers of bone turnover in those patients. In general, BP treatment suppressed biochemical markers of bone turnover, although in some individuals these changes were not statistically significant (Figure 7B).

## Discussion

We show that DKK-1 is a direct target of the mevalonate pathway (Figure 7C). As an inhibitor of osteoblast differentiation and function, elevated DKK-1 levels are thought to contribute to the imbalance of osteoblastic bone formation and osteoclastic bone resorption, a predominant feature in osteolytic bone disease [21]. DKK-1 is best investigated in myeloma bone disease [22] and a monoclonal antibody, BHQ880, directed against DKK-1 is currently being evaluated in a combined phase I/II trial [23].

The role of DKK-1 in breast cancerand prostate cancer is less well characterized. In breast cancer, where predominantly osteolytic lesions are found, a mechanism similar to that proposed in multiple myeloma is plausible. In this study, we identified an increased expression of DKK-1 in hormone-negative and highly osteotropic breast cancer cell lines. This subtype is particularly associated with an unfavorable outcome and a high risk of recurrence. Therefore, adjuvant treatments decreasing the risk of relapse are warranted. Assessment of primary tissue revealed an overexpression of DKK-1 in ER-negative breast cancer cases, but the expression appears independent of tumor grade or stage. Moreover, our data show that breast cancer-derived DKK-1 potently inhibits osteoblast differentiation, as seen assessed by ALP expression, as well as osteoblast-derived production of OPG, a potent inhibitor of osteoclast activity. Zoledronic acid prevented this osteoblast inhibition by negative regulation of DKK-1. This novel mechanism may help to consolidate bone turnover in the metastatic process and provides a rationale to further explore the adjuvant use of BPs in patients with high DKK-1 levels.

In contrast to breast cancer, bone metastases from prostate cancers are predominantly osteosclerotic [24]. Here, the role of DKK-1 is less obvious. DKK-1 levels have been described to increase early in disease development, followed by a decrease with tumor progression and bone metastases, which may depict the molecular switch that transitions the osteolytic phenotype to an osteoblastic phenotype [17]. This concept is supported by the finding that overexpression of DKK-1 in osteoblastic prostate cancer cells induces an osteolytic phenotype [25]. In line with these findings, only the osteolytic PC3 cells expressed relevant levels of DKK-1 in this report. Lowering DKK-1 levels may have direct practical implications for patients with osteotropic tumors because osteoblast activity may be normalized directly and indirectly via OPG and receptor activator of nuclear factor-κB ligand signaling. Silencing of DKK-1 delayed the development of bone lesions in a model of prostate cancer [26], and neutralizing DKK-1 antibodies have been successfully applied to prevent bone lesions in a preclinical model of myeloma bone disease [13]. Besides the promising effects of DKK-1 inhibition on bone metastases, inhibiting DKK-1 also bears the potential risk of promoting tumor proliferation as a result of activated Wnt signaling and a number of studies have defined DKK-1 as a tumor suppressor [27-29]. However, these studies focused on effects of overexpressing DKK-1 mainly in hormone receptor-positive breast cancer cells with low or undetectable baseline levels of DKK-1, and these results may not be transferable to other breast cancer subtypes and other entities. Indeed, DKK-1-overexpressing prostate cancer cells were recently reported to have an increased subcutaneous tumor mass upon ectopic transplantation and a higher incidence of bone metastases after intracardiac injection [30]. Furthermore, genetic disorders of dysfunctional Wnt inhibitors, such as van Buchem’s disease and sclerosteosis, are not associated with an increased risk of malignancies [31]. However, there are no clinical data available to assess the effects of inhibiting Wnt inhibitors (DKK-1 or sclerostin) in patients with existing malignancies, and the potential risk of aggravation should be considered.

In our study, inhibition of the mevalonate pathway resulted in a profound suppression of DKK-1 *in vitro* and in breast cancer patients receiving zoledronic acid. We have previously shown that similar concentrations of zoledronic acid are sufficient to induce apoptosis in treated cancer cells [32,33]. Furthermore, decreased vascular endothelial growth factor levels have been reported in women receiving zoledronic acid [34]. Recent results from two large clinical trials have yielded inconclusive results regarding the general use of zoledronic acid as an adjuvant therapy for breast cancer patients [6,7]. While especially postmenopausal women appeared to benefit from early treatment with zoledronic acid, exact characteristics of these groups remain elusive. More recently, a meta-analysis of three placebo-controlled trials underlined better survival for zoledronic acid treatment of patients with bone metastases from solid tumors who displayed poor prognosis features such as elevated NTX levels as a surrogate for aggressive bone lesions [35]. Here, we found an overexpression of DKK-1 in ER-negative breast cancer patients. This is in line with another study, which reported a relative increase of DKK-1 in ER/progesterone receptor-negative cancer [15]. In our patients, baseline levels of DKK-1 largely varied. This limits the use of DKK-1 as a potential screening marker. Also, it remains unclear whether increases in DKK-1 during the course of a disease are associated with tumor progression. Elevated DKK-1 levels are described in breast cancer patients with confirmed bone lesions [14] and a recent study defined high levels of DKK-1 as a negative prognostic marker in triple-negative breast cancer [16]. It is therefore feasible that lowering DKK-1 levels may have positive effects. Hence, patients with high baseline DKK-1 levels may especially benefit from early therapy with zoledronic acid or potentially another mevalonate pathway inhibitor. We found DKK-1 levels to remain effectively suppressed for at least 12 months. Whereas zoledronic acid is a standard agent for the treatment of established bone metastases, statins are not used in this context. *In vitro*, considerably lower concentrations of atorvastatin than zoledronic acid were needed to achieve a comparable suppression of DKK-1. However, whether the use of statins also translates into a clinically relevant suppression of DKK-1, as seen with zoledronic acid, remains unclear.

Our study has potential limitations. The number of patients included in this study is small and we have no available survival data. Larger clinical trials are therefore needed to fully define the prognostic value and pathophysiological role of DKK-1 in breast cancer. Furthermore, the tissue or cellular source of DKK-1 suppression remains unclear. Women received zoledronic acid as an adjuvant treatment and were tumor free. The drop in DKK-1 is unlikely to stem from the removal of the tumor, because there was no decrease in the placebo-treated group. Bone markers in the zoledronic acid-treated patients are suppressed as expected following antiresorptive treatment. The decrease in serum DKK-1 may therefore be explained by an impaired osteoblastic activity. Alternatively, since cells of vascular origin are also sensitive to zoledronic acid, DKK-1 suppression may also be a cumulative effect derived from multiple cell types.

Furthermore, the doses required to suppress DKK-1 *in vitro* probably exceed achievable serum concentrations *in vivo*. Serum concentrations of zoledronic acid decline rapidly after infusion and, although we have shown that a short exposure time of 2 hours is sufficient to mimic the effects of a continuous BP exposure on DKK-1, it remains unclear whether the observed effects in breast cancer cells *in vitro* can be translated into the clinical setting. BP uptake *in vitro* is likely to be mediated by fluid phase endocytosis and the cells’ ability to interfere with the mevalonate pathway in cellular targets outside the bone is likely to be limited by availability and other pharmacokinetic properties.

Currently, it remains unclear to what extent the DKK-1 serum pool in breast cancer patients affected by bone metastasis is derived from tumor cells versus reactively enhanced bone remodeling and whether the suppression of DKK-1 by zoledronic acid has a beneficial effect on osteoblast/osteoclast activity at the site of an established bone lesion or might even prevent the establishment of disseminated tumor cells in the bone.

## Conclusion

We show that zoledronic acid and atorvastatin, two clinically approved inhibitors of the mevalonate pathway, effectively inhibit the Wnt inhibitor DKK-1 in breast cancer cells and, thereby, prevent the inhibition of WNT3A-induced OPG production in osteoblasts *in vitro*. These findings warrant further studies on DKK-1 in osteolytic bone disease and mevalonate pathway inhibitors in breast cancer.

## Abbreviations

ALP: alkaline phosphatase; BP: bisphosphonate; DKK-1: Dickkopf-1; ER: estrogen receptor; FPP: farnesyl pyrophosphate; GAPDH: glyceraldehyde 3-phosphate dehydrogenase; GGPP: geranyl-geranyl-pyrophosphate; OPG: osteoprotegerin; PCR: polymerase chain reaction; siRNA: small interfering RNA.

## Competing interests

The authors have received grants or honorarium for advisory boards or lectures for the individual or for the institution from Amgen (TDR, LCH, PH, FJ), AstraZeneca (PH), Eli Lilly (PH, FJ), GlaxoSmithKline (PH), Novartis (TDR, LCH, PH, FJ), Pfizer (PH), Roche (PH, FJ), Servier (LCH, FJ), Merck (LCH, TDR, FJ), and Nycomed (LCH, FJ). The remaining authors declare that they have no competing interests.

## Author contributions

TDR and MR designed and performed the experiments, analyzed the data and wrote the paper. AG, ST and PB-M performed the experiments and substantially helped draft the work. PH and TB provided serum samples, performed serum assessment and critically reviewed the manuscript. MHM and GBB conducted the pathology assessment and helped write the paper. FJ, RE, MB and CS helped with experiments and writing of the paper. LCH contributed to the design of the study and writing of the paper. All authors read and approved the final manuscript.

## Supplementary Material

Additional file 1: Figure S1 (A) results from a Wnt signaling pathway PCR array conducted with MDA-231 breast cancer cells after 24 hours of treatment with zoledronic acid. Genes with >2-fold regulation are black. (B) Baseline expression of DKK-1 in osteolytic PC3 and osteoblastic MDA-PCa 2b prostate cancer cells. PC3 cells treated with zoledronic acid (100 μM) or atorvastatin (10 μM) for 24 hours. (C), (D) Human microvascular endothelial cells-1 and human umbilical vein endothelial cells after 24 hours with zoledronic acid (100 μM), atorvastatin (10 μM), GGTI-298 (5 μM) or FTI-277 (100 nM). PCR data presented as the mean ± standard deviation (SD) of three independent experiments. **Figure S2**. MDA-231 cells were exposed to increasing doses of zoledronic acid (A) or atorvastatin (B) for 24 hours. (C) MDA-231 cells were exposed to zoledronic acid for 2 hours. After 2 hours, media were removed, cells were washed twice and fresh media were added. After an additional 24 hours RNA was isolated and DKK-1 expression was assessed. Data are presented as the mean ± SD of three independent experiments. **Figure S3**. (A) validation of DKK-1 antibody staining. MDA-231 cells treated with DKK-1 siRNA or control siRNA, pelleted and embedded in paraffin. Cells were stained with the DKK-1 antibody used for the tissue microarray. No staining was detectable in MDA-231 cells with depleted DKK-1, whereas a strong staining was detectable in control siRNA-treated cells. (B) Patient characteristics of control and breast cancer cohort assessed in Figure 2C. Patients were matched for age, size, weight, menarche, and menopausal status. **Figure S4**. (A) patient characteristics of Figure 7, and (B) individual absolute and relative DKK-1 values of breast cancer patients receiving adjuvant zoledronic acid or placebo every 3 months. Serum DKK-1 was measured at baseline, 6 and 12 months. Mean values of placebo and zoledronic acid treated patients are shown in Figure 7A. Click here for file
