# Supplementary material for: Dickkopf-1 is regulated by the mevalonate pathway in breast cancer
Source: Breast Cancer Res. 2014 Feb 14;16(1):R20. doi: 10.1186/bcr3616 (PMC3979025; doi:10.1186/bcr3616)
Supplement: Additional file 1: Figure S1 — (A) results from a Wnt signaling pathway PCR array conducted with MDA-231 breast cancer cells after 24 hours of treatment with zoledronic acid. Genes with >2-fold regulation are black. (B) Baseline expression of DKK-1 in osteolytic PC3 and osteoblastic MDA-PCa 2b prostate cancer cells. PC3 cells treated with zoledronic acid (100 μM) or atorvastatin (10 μM) for 24 hours. (C), (D) Human microvascular endothelial cells-1 and human umbilical vein endothelial cells after 24 hours with zoledronic acid (100 μM), atorvastatin (10 μM), GGTI-298 (5 μM) or FTI-277 (100 nM). PCR data presented as the mean ± standard deviation (SD) of three independent experiments. Figure S2. MDA-231 cells were exposed to increasing doses of zoledronic acid (A) or atorvastatin (B) for 24 hours. (C) MDA-231 cells were exposed to zoledronic acid for 2 hours. After 2 hours, media were removed, cells were washed twice and fresh media were added. After an additional 24 hours RNA was isolated and DKK-1 expression was assessed. Data are presented as the mean ± SD of three independent experiments. Figure S3. (A) validation of DKK-1 antibody staining. MDA-231 cells treated with DKK-1 siRNA or control siRNA, pelleted and embedded in paraffin. Cells were stained with the DKK-1 antibody used for the tissue microarray. No staining was detectable in MDA-231 cells with depleted DKK-1, whereas a strong staining was detectable in control siRNA-treated cells. (B) Patient characteristics of control and breast cancer cohort assessed in Figure 2C. Patients were matched for age, size, weight, menarche, and menopausal status. Figure S4. (A) patient characteristics of Figure 7, and (B) individual absolute and relative DKK-1 values of breast cancer patients receiving adjuvant zoledronic acid or placebo every 3 months. Serum DKK-1 was measured at baseline, 6 and 12 months. Mean values of placebo and zoledronic acid treated patients are shown in Figure 7A. [file bcr3616-S1.pptx]

## Slide 1
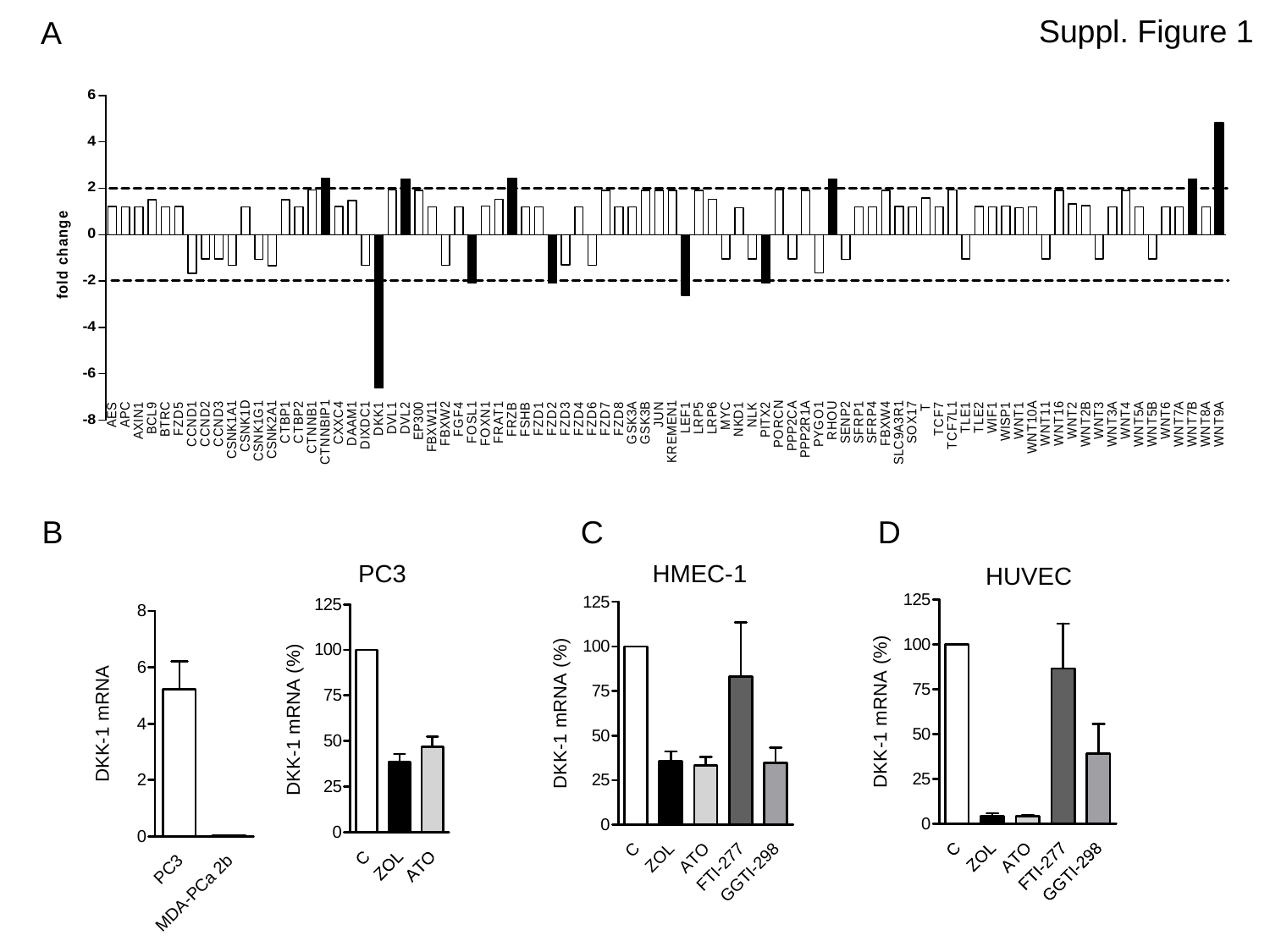

Suppl. Figure 1
A
B
C
D
PC3
HMEC-1
HUVEC

## Slide 2
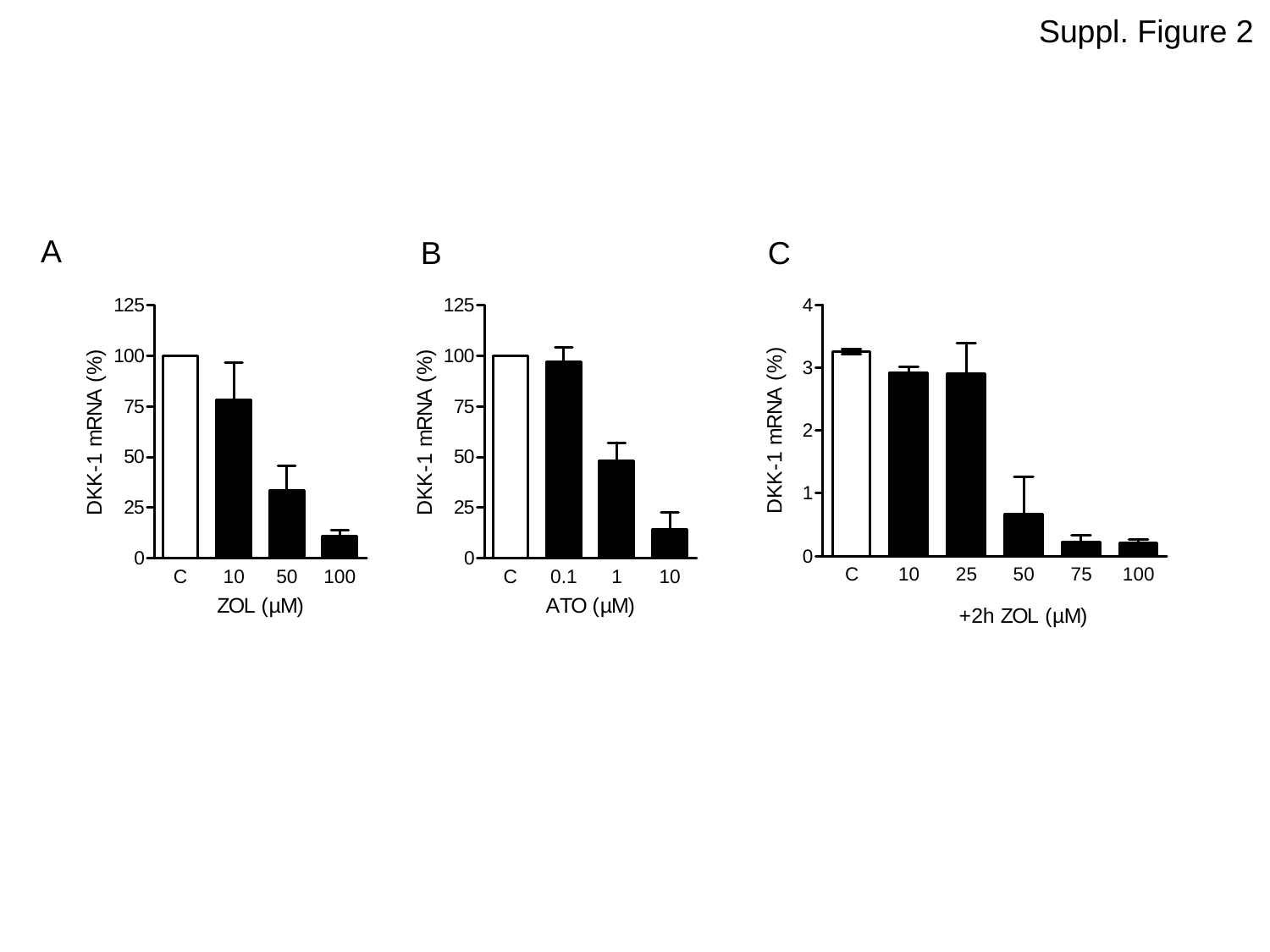

Suppl. Figure 2
A
B
C

## Slide 3
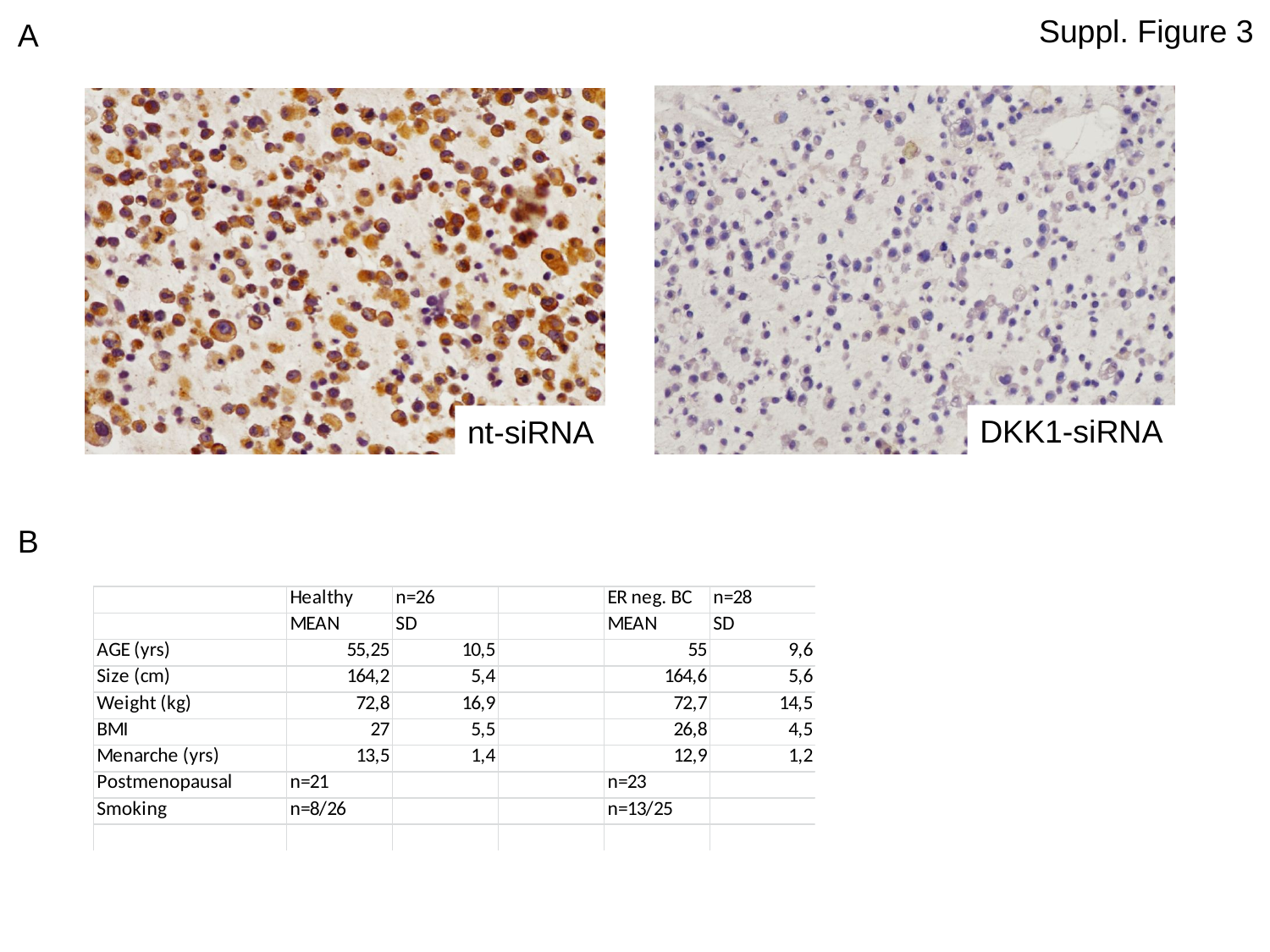

Suppl. Figure 3
A
DKK1-siRNA
nt-siRNA
B

## Slide 4
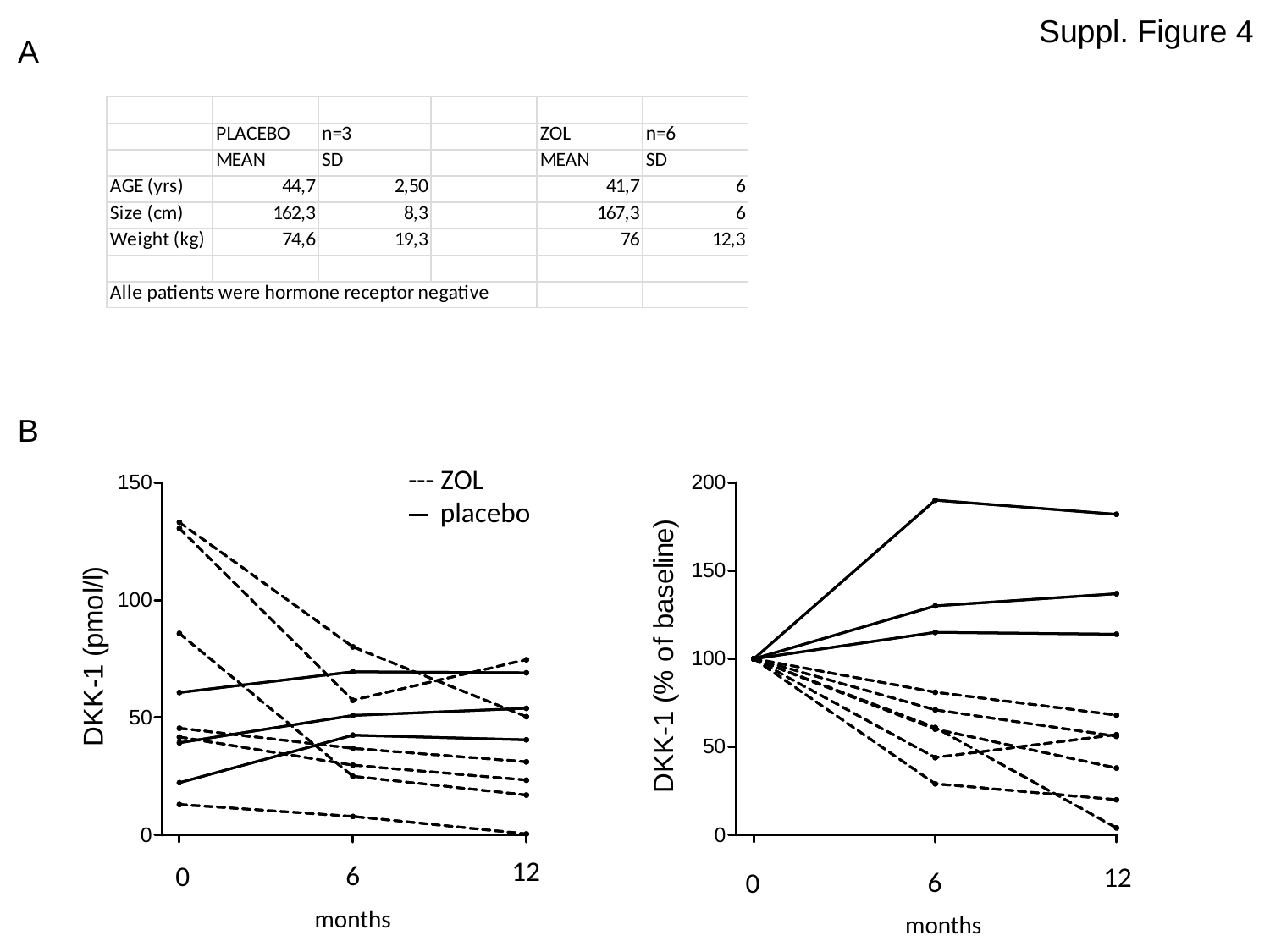

Suppl. Figure 4
A
B
--- ZOL
 placebo
12
6
0
12
6
0
months
months
